# SAPHO syndrome and pustular skin diseases: shared inflammatory circuits, divergent tissue outcomes, and the limits of a spectrum model

**DOI:** 10.3389/fimmu.2026.1906728

**Published:** 2026-08-11

**Authors:** Rongzhen Xia, Kaining Gao, Yishu Zhao, Yiting Lin, Qingrui Yang, Wei Xu

**Affiliations:** 1 Department of Rheumatology and Immunology, Shandong Provincial Hospital Affiliated to Shandong First Medical University, Jinan, Shandong, China; 2 State Key Laboratory of Neurology and Oncology Drug Development, Nanjing, China

**Keywords:** acrodermatitis continua of Hallopeau, autoinflammatory keratinization diseases, generalized pustular psoriasis, Il-23/Th17 axis, IL-36, neutrophils, palmoplantar pustulosis, SAPHO syndrome

## Abstract

Both SAPHO (synovitis, acne, pustulosis, hyperostosis, and osteitis) syndrome and pustular skin diseases are characterized by neutrophil-dominated inflammatory responses; however, whether they represent manifestations within a shared disease spectrum remains unresolved. The aim of this review was not simply to establish equivalence between these conditions but rather to clarify the extent of their overlap, evaluate the strength of current evidence, and discuss the clinical implications of their potential relationship. In this narrative review, we compare SAPHO syndrome with major pustular skin phenotypes, including palmoplantar pustulosis (PPP), generalized pustular psoriasis (GPP), and acrodermatitis continua of Hallopeau (ACH), focusing on clinical manifestations, genetic susceptibility, immune mechanisms, tissue-specific pathological features, and therapeutic responses. The most stable area of overlap between SAPHO syndrome and pustular skin diseases is observed within the SAPHO–PPP subgroup, whereas direct evidence supporting equivalence between SAPHO syndrome and GPP or ACH remains limited. IL-36 signaling, the IL-23/Th17 axis, keratinocyte activation, myeloid cell responses, and neutrophil recruitment represent potentially shared downstream inflammatory networks. However, although more direct evidence has demonstrated IL-36 pathway dysregulation in a subset of patients with GPP, its contribution to SAPHO-associated osteitis remains unvalidated at the tissue level. The characteristic features of SAPHO syndrome, including aseptic osteitis, hyperostosis, preferential anterior chest wall involvement, and chronic pathological bone remodeling, also differ from the osteoarticular manifestations observed in most pustular skin diseases. Therefore, we propose that SAPHO syndrome and specific pustular phenotypes should be regarded as related but distinct disorders within an overlapping inflammatory network, rather than as different manifestations of a single unified disease entity. This framework may improve interdisciplinary recognition and provide a basis for mechanism-oriented research and stratified therapeutic hypotheses; however, it should not yet be considered a substitute for diagnostic or therapeutic algorithms validated through prospective, tissue-resolved, and mechanism-driven studies.

## 1 Introduction

SAPHO syndrome is a rare inflammatory disorder that involves multiple medical disciplines, including rheumatology, dermatology, and radiology. Its name encompasses the five characteristic clinical manifestations: synovitis, acne, pustulosis, hyperostosis, and osteitis (1). Clinically, patients may present with pain involving the anterior chest wall, spine, sacroiliac joints, mandible, or peripheral skeleton. Alternatively, some patients may initially seek dermatological evaluation due to palmoplantar pustulosis, severe acne, or other neutrophil-mediated skin manifestations (2, 3). Because cutaneous and osteoarticular manifestations often develop asynchronously, SAPHO syndrome is frequently associated with delayed diagnosis in clinical practice (4, 5).

Pustular dermatoses do not represent a single disease entity. Generalized pustular psoriasis (GPP) is characterized by acute or subacute episodes of generalized sterile pustules accompanied by systemic inflammation and may lead to organ involvement in severe cases (6, 7). Palmoplantar pustulosis (PPP) typically presents as chronic, localized, and recurrent sterile pustules affecting the palms and soles, with associations with smoking, alterations in local epithelial barrier function, and distinct genetic backgrounds (8, 9). Acrodermatitis continua of Hallopeau (ACH) predominantly affects the distal digits and may result in nail unit destruction and focal osteolytic changes (10). Although these disorders share the morphological feature of pustule formation, they differ substantially in epidemiological characteristics, genetic architecture, disease course, and risk of systemic involvement (11, 12).

This heterogeneity raises a central question addressed in this review: when patients with SAPHO syndrome present with PPP, GPP-like lesions, or other pustular phenotypes, should these manifestations be interpreted as cross-organ manifestations of a single disease process, coincidental coexistence of two distinct conditions, or overlapping patient subgroups that share certain inflammatory pathways but exhibit different tissue-specific outcomes? This question has direct implications for diagnostic classification, imaging strategies, targeted therapeutic selection, and inclusion criteria for clinical studies.

Previous discussions have often considered SAPHO syndrome, pustular psoriasis, and psoriatic arthritis separately, which may lead to confusion between shared cytokine pathways and shared disease origins. The distinctive focus of this review is to place the SAPHO–PPP interface at the center of analysis while incorporating GPP and ACH as comparative pustular phenotypes. We evaluate evidence supporting or challenging a disease-spectrum interpretation across different levels of biological and clinical evidence, and we distinguish conclusions that may inform clinical recognition from hypotheses that remain to be validated.

**Figure 1:**
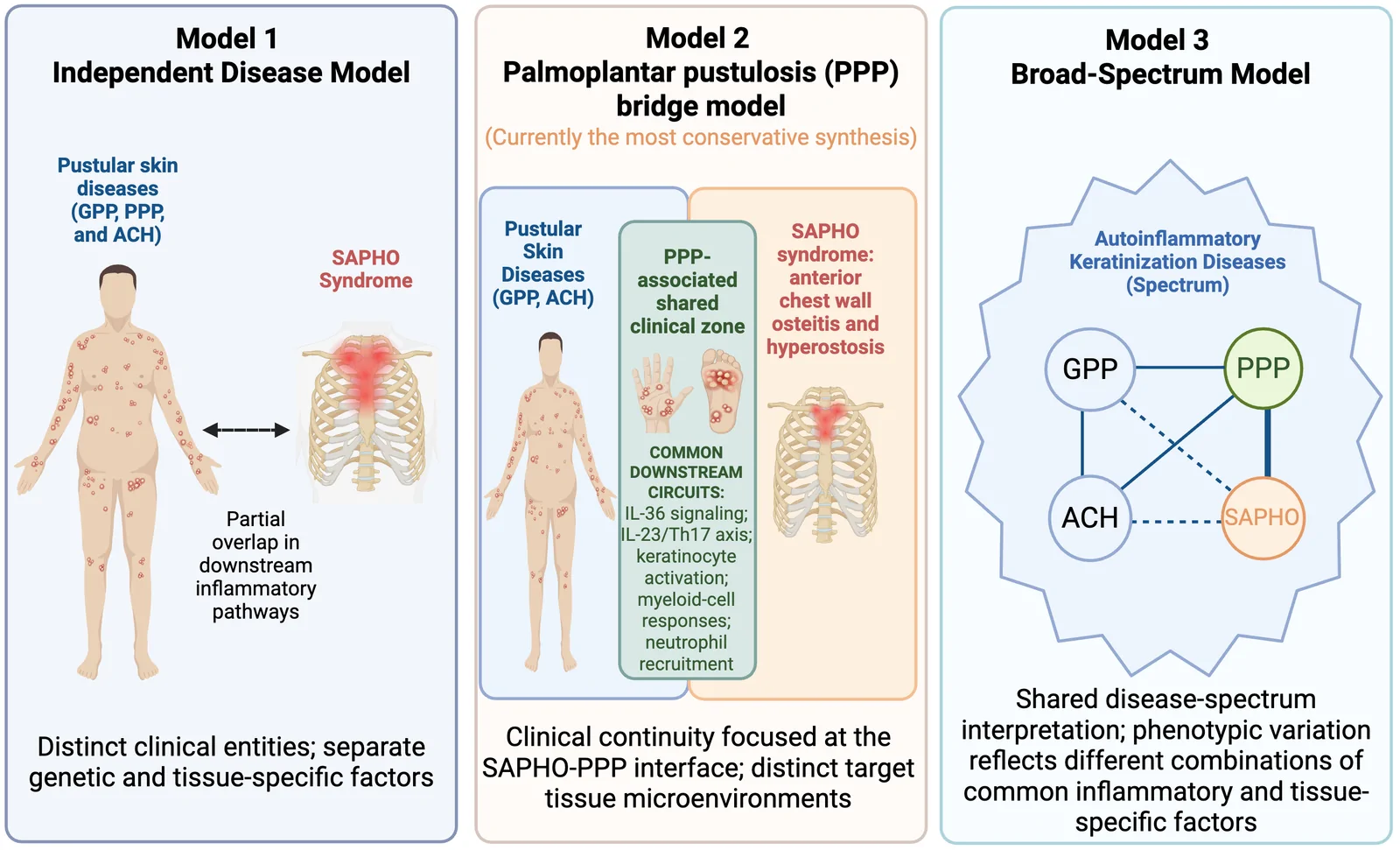
Competing classification models linking SAPHO syndrome with pustular skin diseases. SAPHO, synovitis, acne, pustulosis, hyperostosis, and osteitis.

Accordingly, this review critically examines the relationship between SAPHO syndrome and major pustular skin phenotypes, including PPP, GPP, and ACH. Rather than presuming that these conditions belong to a single disease spectrum, we critically evaluate existing evidence from multiple perspectives, including clinical characteristics, genetics, immunology, histopathology, and therapeutic responses. Three competing conceptual models are discussed: (1) the independent disease model with partial overlap, (2) the PPP-centered bridging model, and (3) the broader autoinflammatory keratinization disorder spectrum model (**Figure 1**). The value of this review lies not in demonstrating a shared etiology between SAPHO syndrome and pustular skin diseases but in defining the boundaries of SAPHO–PPP overlap, distinguishing well-supported evidence from untested hypotheses, and providing a testable framework for future tissue-resolved studies and organ-specific therapeutic evaluation.

## 2 Methods

This narrative review examined the clinical and immunological relationships between SAPHO syndrome and pustular skin diseases. PubMed was searched from database inception to May 1, 2026. The reference lists of relevant reviews and key original studies were also examined to identify additional publications that were not retrieved through the initial search. Because this review was designed as a narrative rather than a systematic review, the Preferred Reporting Items for Systematic Reviews and Meta-Analyses (PRISMA) framework was not applied, no review protocol was registered, and study selection and risk-of-bias assessment were not conducted as formal systematic review procedures.

The search used combinations of disease-related and mechanism-related terms. Disease-related terms included “SAPHO syndrome”, “synovitis acne pustulosis hyperostosis osteitis”, “palmoplantar pustulosis”, “generalized pustular psoriasis”, “acrodermatitis continua of Hallopeau,” “pustulotic arthro-osteitis,” and “psoriatic arthritis”. These terms were combined, where appropriate, with “IL-36”, “IL36RN”, “IL-23”, “Th17”, “IL-17”, “neutrophil”, “osteitis”, “hyperostosis”, “bone remodeling”, “genetics”, “imaging”, “biologic therapy”, and “targeted therapy”.

Peer-reviewed original studies, clinical trials, cohort studies, case series, clinically informative case reports, and relevant reviews were considered. Articles were selected if they provided information on clinical overlap, genetic susceptibility, immune mechanisms, histopathology, imaging findings, or organ-specific treatment responses in SAPHO syndrome, PPP, GPP, ACH, or pustulotic arthro-osteitis. Studies of psoriasis, psoriatic arthritis, and other inflammatory disorders were included only when they provided mechanistic information directly relevant to the pathways discussed in this review.

Studies were not considered when they failed to distinguish among pustular disease phenotypes, focused exclusively on plaque psoriasis without clear relevance to pustular or osteoarticular disease, lacked sufficient clinical or mechanistic information, or were available only as conference abstracts without verifiable data. Only English-language publications with accessible bibliographic and scientific information were included.

Evidence was interpreted according to its disease and tissue specificity. Findings obtained directly from patients with SAPHO syndrome, particularly studies of osteitis lesions or well-defined SAPHO–PPP cohorts, were given the greatest weight when discussing SAPHO pathogenesis. Evidence from GPP, PPP, psoriatic arthritis, other inflammatory diseases, animal models, and *in vitro* experiments was used to assess biological plausibility but was not regarded as direct evidence of SAPHO-associated bone disease. Treatment findings were also considered separately according to study design and organ-specific outcomes, with clinical trials and cohort studies distinguished from case reports.

No quantitative synthesis was undertaken. The evidence was organized around clinical phenotypes, tissue-specific inflammatory mechanisms, bone remodeling, treatment responses, and competing disease-classification models.

## 3 Clinical phenotypes: overlap is primarily centered on the SAPHO–PPP interface

### 3.1 Pustular skin phenotypes and classification boundaries

Clinical phenotype provides the first level of evidence for determining whether two conditions have a genuine disease-spectrum relationship. If two diseases share several inflammatory pathways but lack a stable and reproducible pattern of clinical co-occurrence, they should not be prematurely classified as manifestations of the same disease entity. At the phenotypic level, the association between SAPHO syndrome and PPP is the most consistently observed (13).

Cutaneous lesions represent the most readily recognizable manifestation of overlap between SAPHO syndrome and pustular skin diseases. PPP is the most common form of skin involvement in SAPHO syndrome (14). Histopathologically, PPP is characterized by intraepidermal accumulation of neutrophils with pustule formation, whereas clinically, it presents with recurrent sterile pustules, erythema, scaling, fissuring, and pain affecting the palms and soles (10). In patients with suspected SAPHO syndrome, a current or previous history of PPP remains an important diagnostic clue, even when the cutaneous lesions are inactive at the time of presentation (2, 13).

PPP may coexist with osteitis or pustulotic arthro-osteitis (15), forming a clinically meaningful skin–bone association pattern (13, 16). In contrast, comparably robust epidemiological, genetic, and histopathological evidence directly linking SAPHO syndrome to GPP is currently lacking (17). Therefore, findings derived from studies of IL36RN-associated GPP, including the rapid response to IL-36 receptor blockade, should not be extrapolated to SAPHO-associated bone disease without direct validation (6).

### 3.2 Osteoarticular phenotypes and structural outcomes

Osteoarticular involvement is a defining feature that distinguishes SAPHO syndrome from most pustular skin diseases. Its main pathological manifestations include aseptic osteitis, hyperostosis, synovitis, enthesitis, and chronic pathological bone remodeling (18). Anterior chest wall involvement is particularly characteristic, most commonly affecting the sternoclavicular joints, manubrium, medial ends of the clavicles, and first sternocostal regions (14, 19). Bone scintigraphy may reveal the characteristic “bull’s head sign” (4). The spine, sacroiliac joints, mandible, and long bones may also be involved (14, 20). MRI is useful for detecting active inflammatory changes, particularly bone marrow edema (2), whereas CT is more sensitive for identifying osteosclerosis, hyperostosis, and other proliferative osseous changes (4, 14).

Enthesitis may occur alongside osteitis and hyperostosis, particularly in the anterior chest wall and paraspinal regions (5). Emerging imaging modalities, such as ^68^Ga-FAPI-04 PET/CT, may improve the detection of inflammatory lesions enriched in activated fibroblasts; however, their incremental clinical value and standardized acquisition and interpretation protocols remain to be established (21, 22). Microbiological investigations are usually negative, and conventional inflammatory markers lack disease specificity (23, 24). Osteolytic and hyperostotic changes may coexist during the course of the disease. These features indicate that SAPHO syndrome should not be regarded simply as conventional inflammatory arthritis accompanied by skin lesions.

Musculoskeletal involvement associated with pustular psoriasis is generally classified within the spectrum of psoriatic arthritis and may include peripheral arthritis, dactylitis, enthesitis, and axial spondyloarthritis (25). ACH may cause distal bone destruction, whereas PPP is often associated with arthritis or pustulotic arthro-osteitis (16). However, the hyperostosis and anterior chest wall osteitis characteristic of SAPHO syndrome are distinct from the osteoarticular manifestations of GPP and ACH (13, 16). In particular, the focal osteolysis seen in ACH differs from the chronic bone remodeling pattern of SAPHO syndrome, which is dominated by osteosclerosis and hyperostosis.

### 3.3 Disease course and tissue-specific disease expression

The clinical course differs substantially among these conditions. GPP typically follows a fluctuating pattern of acute flares and remission, whereas PPP is characterized by persistent or recurrent skin lesions. By contrast, SAPHO syndrome often presents with chronic bone pain accompanied by intermittent inflammatory activity (5). These differences may reflect both disease-specific mechanisms and the distinct kinetics of inflammation in skin and bone. Keratinocytes and neutrophils can rapidly drive pustule formation, whereas bone remodeling develops over a much longer period. Improvement of cutaneous lesions does not necessarily indicate resolution of osteitis or arrest of structural bone damage (23).

Differences in the local tissue microenvironment may help explain both the phenotypic overlap and divergence among these diseases. In the skin, keratinocytes, sweat gland structures, the epidermal barrier, local microbiota, and infiltrating neutrophils contribute to a pustular inflammatory environment characterized by increased IL-36 activity (26). In the bone marrow, synovium, cortical bone, and entheses, stromal cells, osteoblasts, osteoclasts, and myeloid cells may translate inflammatory signals into bone pain, bone marrow edema, erosion, osteosclerosis, and hyperostosis. Thus, the presence of shared inflammatory cytokines does not imply identical pathological outcomes. The same downstream inflammatory mediators may produce markedly different clinical phenotypes depending on the local cellular environment, disease stage, mechanical loading, and pre-existing tissue damage (27).

### 3.4 Diagnostic implications

In patients with SAPHO syndrome or pustular psoriasis-associated arthropathy, important differential diagnoses include infection, malignancy, chronic recurrent multifocal osteomyelitis, spondyloarthritis, and psoriatic arthritis (2, 28). Acute pustular eruptions should also be distinguished from acute generalized exanthematous pustulosis, infectious pustular dermatoses, subcorneal pustular dermatosis, and drug-induced paradoxical pustular reactions. Acute bone pain accompanied by bone marrow edema on imaging may initially be mistaken for infectious osteomyelitis or bone metastasis and therefore requires careful evaluation (4, 29, 30). In rare cases, SAPHO syndrome may be accompanied by sterile abscesses of the anterior chest wall, which should be differentiated from infectious abscesses (17, 31).

The absence of active skin lesions does not exclude SAPHO syndrome because musculoskeletal symptoms may precede cutaneous manifestations, and some patients report only mild or remote palmoplantar disease (32). Conversely, occult osteitis should be considered in patients with refractory PPP who develop inflammatory anterior chest wall pain, recurrent sternoclavicular swelling, inflammatory back pain, or otherwise unexplained osteosclerosis (29). Skeletal imaging should be guided by symptoms, physical findings, and the estimated likelihood of osteoarticular involvement rather than performed routinely in all patients with pustular skin disease. Multidisciplinary assessment is particularly useful in patients with asynchronous, atypical, or multisystem manifestations. The principal clinical phenotypes and diagnostic boundaries of SAPHO syndrome, PPP, GPP, and ACH are summarized in **Table 1**.

**Table 1:** Clinical phenotypes and diagnostic boundaries of SAPHO syndrome and major pustular skin diseases.

Domains	SAPHO syndrome	PPP	GPP	ACH
Core phenotypes	Aseptic osteitis and hyperostosis, often accompanied by synovitis or enthesitis; may coexist with PPP, severe acne, or other neutrophilic dermatoses.	Chronic or recurrent sterile pustules on the palms and soles, accompanied by erythema, scaling, fissuring, and pain.	Acute or subacute generalized sterile pustules with systemic inflammation; organ involvement may occur in severe cases.	Persistent pustules involving the nail unit and distal digits; nail destruction and focal osteolysis may occur.
Distribution	Predominantly involves the anterior chest wall; the spine, sacroiliac joints, mandible, and long bones may also be affected.	Palms and soles.	Generalized cutaneous involvement.	Distal fingers or toes and nail units.
Course of disease	Usually chronic; cutaneous and osteoarticular activity are often asynchronous.	Usually persistent or recurrent.	Acute flares alternating with remission.	Usually persistent.
Systemic characteristics	Inflammatory markers lack specificity.	Usually confined to the skin.	Systemic inflammation is a defining feature.	Usually localized.
Musculoskeletal characteristics	MRI may show bone marrow edema, whereas chronic lesions may progress to osteosclerosis or hyperostosis.	May be associated with arthritis or pustulotic arthro-osteitis.	When present, musculoskeletal involvement is generally classified within the psoriatic arthritis spectrum.	Distal interphalangeal joint inflammation and focal distal osteolysis may occur.
Diagnostic boundary/relation to SAPHO		Represents the strongest and most reproducible clinical overlap with SAPHO syndrome. Occult osteitis should be considered in refractory PPP accompanied by chest wall pain, sternoclavicular swelling, inflammatory back pain, or unexplained osteosclerosis.	Direct epidemiological, genetic, and tissue-level equivalence with SAPHO syndrome has not been established. GPP should be distinguished from acute generalized exanthematous pustulosis and infectious or drug-induced pustular eruptions.	Focal osteolysis differs from the osteosclerotic and hyperostotic remodeling characteristic of SAPHO syndrome; direct equivalence is not supported.

ACH, acrodermatitis continua of Hallopeau; GPP, generalized pustular psoriasis; MRI, magnetic resonance imaging; PPP, palmoplantar pustulosis; SAPHO, synovitis, acne, pustulosis, hyperostosis, and osteitis.

## 4 Immunopathology: shared inflammatory circuits, uneven evidence, and tissue-specific consequences

### 4.1 Genetic architecture

Genetic studies suggest both convergence and divergence across these disease pathways. Variants in genes such as IL23R and TYK2, which regulate IL-23/Th17 signaling, may partly explain why psoriatic skin disease and some forms of osteoarticular involvement respond to similar targeted therapies (33). However, the strength of these associations, their disease specificity, and their distribution across populations vary considerably. Pathway convergence alone is insufficient to establish that SAPHO syndrome and pustular skin diseases share a common genetic origin (34).

Loss-of-function variants in IL36RN are among the best-established genetic risk factors for certain subtypes of GPP (35, 36). Deficiency of the IL-36 receptor antagonist results in excessive IL-36 signaling, leading to keratinocyte activation, chemokine production, and neutrophil-rich epidermal inflammation (35, 37). Other genes, including CARD14, AP1S3, and MPO, have also been implicated in pustular disease, although their contribution varies across ethnic and geographic populations. Current evidence supports IL-36 dysregulation as a key pathogenic mechanism in a subset of patients with GPP, but comparable genetic evidence has not established abnormal IL-36 signaling as a major cause of SAPHO syndrome (38–40).

The genetic background of SAPHO syndrome appears highly heterogeneous, and the disease is generally considered a complex polygenic disorder (5). In patients with axial involvement or a spondyloarthritis-like phenotype, susceptibility may be associated with HLA-B27 and other variants within the major histocompatibility complex, although these associations differ across populations (29, 41). Abnormal microbial recognition, stimulation by low-virulence organisms such as *Cutibacterium acnes*, and dysregulated myeloid cell responses have also been proposed as contributors to disease development. At present, however, these mechanisms should be regarded as potential pathogenic pathways or disease-modifying factors rather than established genetic causes of SAPHO syndrome (1, 42).

Beyond established genetic associations, Forkhead box O1 (FoxO1) has been proposed as a potential host-response regulator linking impaired bacterial clearance to inflammation in SAPHO syndrome (43, 44). Reduced FoxO1 activity may facilitate the persistence or immune escape of *C. acnes* and subsequently contribute to inflammatory activation involving IL-1β, IL-17, and TNF-α (43, 44). However, FoxO1 has not been established as a SAPHO susceptibility gene through genetic association or SAPHO-specific tissue-level functional studies. Its involvement should be considered a biologically plausible but unconfirmed pathogenic hypothesis rather than a defined genetic determinant of SAPHO syndrome.

### 4.2 IL-36, the IL-23/Th17 axis, and neutrophil-driven amplification

The IL-23/Th17 axis provides substantial evidence of mechanistic convergence across pustular inflammatory diseases. IL-23 promotes the production of IL-17A, IL-17F, and IL-22 by both adaptive and innate lymphocyte populations (45). Within the skin microenvironment, IL-17 family cytokines stimulate keratinocytes to release antimicrobial peptides and chemokines, including CXCL1, CXCL8, and CCL20, thereby promoting the recruitment of neutrophils and inflammatory lymphocytes to lesional skin (13, 46). TNF-α further amplifies this response, whereas IL-22 mainly regulates keratinocyte proliferation and differentiation (47).

IL-36 signaling is also closely linked to pustular inflammation (48). Keratinocyte-derived IL-36 can act in an autocrine manner on neighboring keratinocytes and in a paracrine manner on dendritic and myeloid cells, inducing the production of proinflammatory mediators such as IL-23, IL-1β, IL-6, and CXCL8 (22, 49, 50). Neutrophil-derived proteases can further process and activate IL-36 precursors, while IL-36-induced chemokines recruit additional neutrophils to the lesion, creating a self-reinforcing inflammatory circuit (51–53). In a murine psoriasis model, IL-17A-driven skin inflammation was shown to be critically dependent on IL-36 signaling (50). These findings provide mechanistic plausibility for pustular skin inflammation but should not be regarded as direct evidence of the same pathway operating in SAPHO osteitis. This pathway provides a plausible explanation for the central pathological features of pustular skin disease. Its involvement in SAPHO-associated osteitis is biologically plausible, but direct experimental and clinical evidence remains limited (34, 41, 54).

Other endogenous mediators may further sustain neutrophil-dominated inflammation. S100A8/A9 can function as damage-associated molecular patterns and has been implicated in myeloid cell activation and psoriatic inflammation (55). However, direct evidence defining its contribution to SAPHO osteitis remains limited. Neutrophil extracellular trap formation, neutrophil-derived IL-26, complement activation, and impaired autophagy may also contribute to neutrophil-dominated inflammatory cascades (13, 56, 57).

Culture-based and infection-focused studies have examined *C. acnes* in SAPHO osteitis lesions, but detection rates have been inconsistent, and its causal role remains uncertain (43, 44, 58). In one series of 56 patients with SAPHO syndrome, osteitic bone biopsy was performed in six patients and *C. acnes* was isolated from only one specimen (58). These observations support further investigation of a possible microbial contribution but do not establish SAPHO syndrome as an active bacterial infection. More importantly, paired molecular and immunological analyses of pustular skin and osteitis tissues from the same patients remain scarce. Such studies are needed to determine whether IL-36-, IL-23/Th17-, and neutrophil-related pathways operate similarly across the two tissue compartments.

### 4.3 Bone inflammation and remodeling

IL-17, IL-23, IL-1β, TNF-α, and inflammasome-related signaling can act on synovial cells, bone marrow stromal cells, osteoblast-lineage cells, and osteoclast precursors within osteoarticular tissues (47). Osteoclast differentiation may proceed through both RANKL-dependent and RANKL-independent mechanisms, whereas prolonged exposure to inflammatory mediators can promote aberrant osteoblast activity and pathological bone formation (59).

Early SAPHO lesions may present with bone marrow edema or focal osteolytic changes, whereas chronic lesions are more commonly characterized by osteosclerosis and hyperostosis (14, 60). These cross-sectional imaging and histopathological observations are compatible with a temporal change in bone remodeling patterns. However, progression from an early osteolytic phase to a later osteosclerotic or hyperostotic phase has not been confirmed in prospective longitudinal tissue studies.

Within the local osteoarticular microenvironment, IL-17, TNF-α, and IL-1β act on synovial fibroblasts, bone marrow stromal cells, and osteoblast-lineage cells, shifting bone remodeling from homeostatic coupling toward an inflammation-driven imbalance between bone resorption and bone formation. IL-17 promotes osteoclastogenesis primarily by increasing RANKL expression in osteoblast-lineage cells, synovial fibroblasts, and bone marrow stromal cells, while weakening OPG-mediated inhibition and thereby increasing the RANKL/OPG ratio. It should be noted that much of the evidence linking IL-17 to RANKL-dependent osteoclastogenesis originates from rheumatoid arthritis samples, cellular experiments, and experimental models rather than from SAPHO bone tissue (61–63). Therefore, these findings support a biologically plausible mechanism but do not demonstrate a SAPHO-specific bone remodeling pathway. Under certain inflammatory conditions, IL-17 may also enhance the responsiveness of osteoclast precursors to RANKL (61–63).

Binding of RANKL to RANK activates TRAF6-dependent NF-κB, MAPK/c-Fos, and NFATc1 signaling. NFATc1 is a key transcription factor that drives osteoclast differentiation and the expression of bone-resorption genes, including tartrate-resistant acid phosphatase and cathepsin K (61, 63). TNF-α further enhances the sensitivity of osteoclast precursors to RANKL and acts synergistically with IL-17, IL-1β, and low levels of RANKL to amplify local bone resorption and bone marrow inflammation (64). At the same time, IL-17 and TNF-α stimulate osteoblast-lineage and stromal cells to produce IL-6, prostaglandin E2, macrophage colony-stimulating factor, and several chemokines. Osteoblast-lineage cells therefore function not only as bone-forming cells but also as immunometabolic regulators of osteoclastogenesis and persistent inflammation (62).

One possible interpretation is that early inflammatory or osteolytic activity and later osteosclerotic or hyperostotic features represent different phases of tissue remodeling. However, this biphasic model remains hypothetical because it has not been established in prospective longitudinal tissue studies.

The ability of inflammatory cytokines to promote both osteoclastogenesis and pathological bone formation also shows why disease heterogeneity cannot be explained by cytokine abnormalities alone. Psoriasis, psoriatic arthritis, spondyloarthritis, and SAPHO syndrome share several inflammatory pathways, but these common downstream signals do not explain why some patients primarily develop pustular skin lesions, others erosive arthritis, and others marked hyperostosis. Mechanical stress, enthesis microdamage, stromal-cell programming, disease stage, and the local bone remodeling environment are likely to influence the final tissue phenotype.

### 4.4 An integrated but non-unitary model

**Figure 2:**
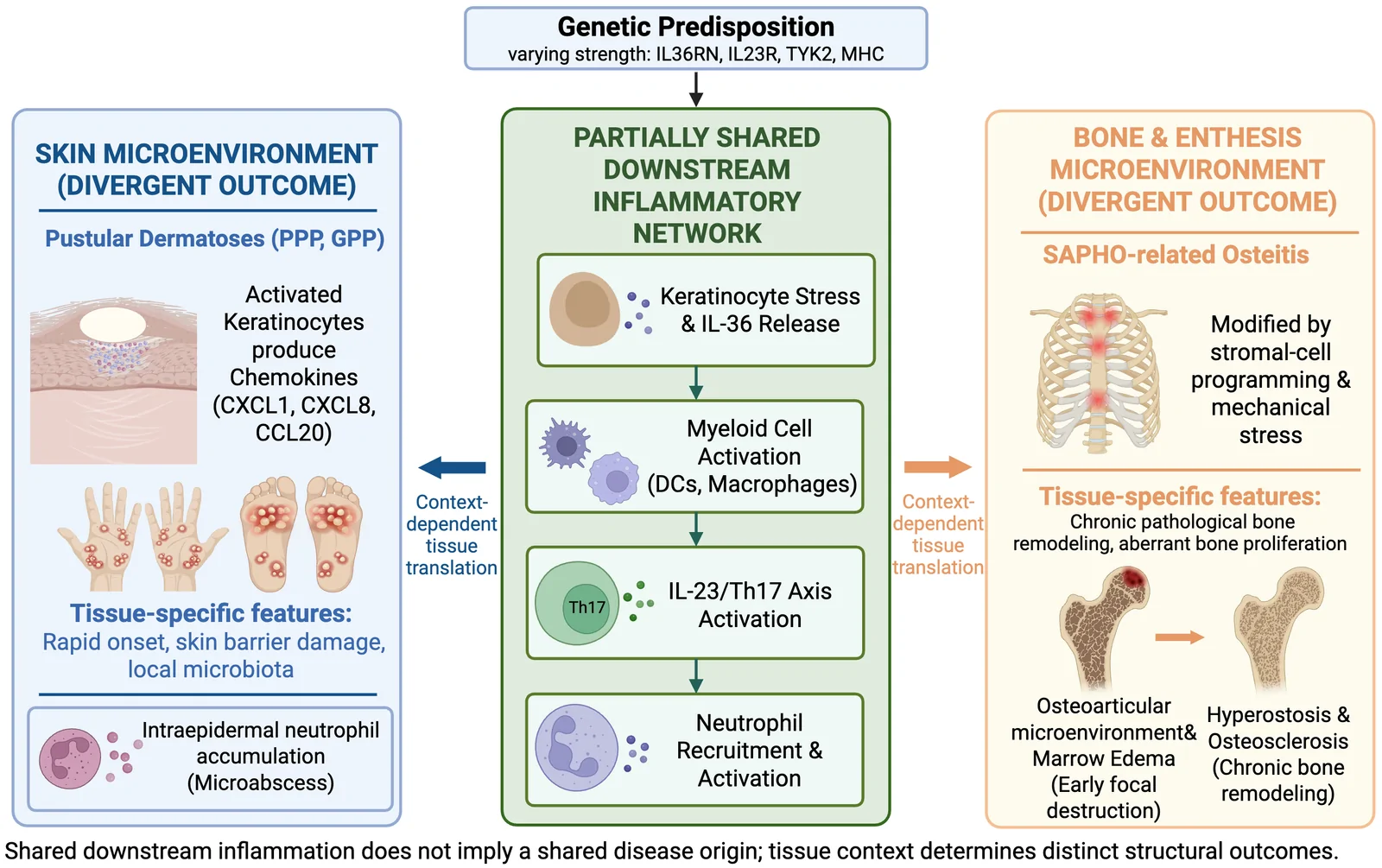
Partially shared inflammatory circuits and divergent tissue outcomes in SAPHO syndrome and pustular skin diseases. SAPHO, synovitis, acne, pustulosis, hyperostosis, and osteitis.

A useful framework should explain the observed clinical overlap while preserving meaningful disease boundaries. Current evidence supports an inflammatory cellular network involving keratinocytes, myeloid cells, dendritic cells, Th17 cells, γδ T cells, and neutrophils across pustular psoriasis-related conditions. Disease heterogeneity may arise at three interrelated levels. First, genetic and epigenetic factors may determine whether IL-36, IL-23/Th17, MHC-related signaling, or other pathways predominate. Second, the local microenvironments of the skin, entheses, bone marrow, synovium, and cortical bone may shape distinct inflammatory responses, leading to pustules, arthritis, osteitis, osteosclerosis, or hyperostosis. Third, environmental and treatment-related factors, including smoking, infection, drug exposure, gut microbiota dysbiosis, and mechanical injury, may trigger or aggravate disease activity (65). **Figure 2** summarizes how partially shared downstream inflammatory circuits may give rise to different cutaneous and skeletal phenotypes.

This model explains the clinical relevance of overlapping phenotypes without requiring SAPHO syndrome and pustular skin diseases to be classified as a single disease entity. It also generates a testable hypothesis: among patients with SAPHO syndrome, those with active PPP may show greater IL-36 pathway activation and neutrophil infiltration in skin lesions than those without cutaneous involvement. By contrast, abnormal bone remodeling may remain a defining feature of osteitis regardless of the presence of skin disease.

The competing classification models and inflammatory network presented in this review are intended as hypothesis-generating frameworks. They distinguish relatively well-supported observations from questions that still require validation and should not be interpreted as established disease-spectrum models or as clinical diagnostic and therapeutic algorithms.

## 5 Treatment response: supportive but inconclusive translational evidence

### 5.1 Conventional therapies and discordant organ responses

Commonly used treatments include non-steroidal anti-inflammatory drugs (NSAIDs), bisphosphonates, antibiotics, glucocorticoids, methotrexate, cyclosporine, acitretin, iguratimod, and other immunosuppressive agents (14, 66). NSAIDs and bisphosphonates may relieve bone pain and reduce osteitis activity in some patients with SAPHO syndrome (14, 59). Short courses of NSAIDs combined with glucocorticoids may help control acute inflammation (67), and improvement of SAPHO-associated pleural effusion has also been reported (4, 68). Acitretin, cyclosporine, and methotrexate may be more effective for pustular skin lesions (69). Overall treatment responses vary considerably between patients (24), relapses are common (70), and a therapy that benefits one organ system may have limited effects on another (34, 71). This organ-specific discordance argues against assuming that cutaneous and skeletal lesions are driven by identical mechanisms.

Treatment response can inform mechanistic hypotheses, but the underlying evidence must be interpreted cautiously. Several targeted therapies have been evaluated in clinical trials for GPP, whereas evidence for biologic agents and small-molecule therapies in SAPHO syndrome is derived largely from retrospective cohorts, small case series, and individual case reports (71). Treatment decisions should consider the dominant clinical phenotype, the organs involved, the strength of available evidence, and potential adverse effects, rather than extrapolating efficacy from one organ system to another.

### 5.2 Targeting the IL-36 pathway

IL-36 receptor inhibitors have demonstrated efficacy in acute GPP (72). Spesolimab rapidly improves pustular eruptions and systemic inflammation by interrupting the IL-36-mediated inflammatory circuit between keratinocytes and myeloid cells (39, 73). These clinical findings provide important pharmacological support for the central role of IL-36 signaling in GPP pathogenesis (60, 74).

By contrast, evidence for IL-36 receptor blockade in SAPHO syndrome remains limited. In patients with SAPHO syndrome who develop acute GPP-like flares or have a genetically defined IL-36-related pustular phenotype, IL-36 inhibition may be biologically plausible (26). However, there is currently insufficient evidence that it improves osteitis, hyperostosis, or chronic bone pain (39, 74, 75). A pattern in which pustular lesions resolve rapidly while bone marrow edema on MRI and bone pain persist would suggest partial pathway overlap rather than a shared disease process (56). Therefore, IL-36 inhibitors should be regarded as an exploratory option for selected overlap phenotypes rather than as a standard treatment for SAPHO-associated osteitis.

### 5.3 Targeting the IL-23/Th17 axis

Biologic agents targeting IL-12/23 p40, IL-23 p19, IL-17A, IL-17A/F, and IL-17RA are widely used in the treatment of psoriasis and psoriatic arthritis (76, 77). Case reports and observational studies suggest that some patients with SAPHO syndrome and concomitant PPP may experience improvements in both cutaneous and osteoarticular manifestations after blockade of the IL-23/Th17 axis (15, 34). These responses support the possibility of shared downstream inflammatory pathways, but do not establish identical initiating mechanisms in the two conditions (59, 78).

More specifically, two case reports have described coordinated improvement of cutaneous and musculoskeletal manifestations following treatment with risankizumab, a selective IL-23p19 inhibitor. Flora et al. reported rapid and sustained improvement in a patient with SAPHO syndrome and prominent PPP, although concomitant colchicine and corticosteroid treatment and limited objective assessment of skeletal disease complicated attribution of the response to risankizumab alone (79). Ferraioli et al. subsequently described a patient with bilateral knee synovitis, PPP, and guttate psoriasis who achieved clinical remission by week 24 and maintained the response through week 52. Improvements were reported in Disease Activity Index for Psoriatic Arthritis (DAPSA), Psoriasis Area and Severity Index (PASI), ultrasonographic synovitis, and health-related quality of life (80). These cases provide preliminary clinical support for possible IL-23 pathway involvement in selected SAPHO–PPP phenotypes. Nevertheless, both are uncontrolled single-patient observations and do not establish efficacy for chronic osteitis, hyperostosis, or structural bone progression.

The broad biological functions and tissue-specific effects of IL-17 signaling also require consideration (81). Bimekizumab neutralizes both IL-17A and IL-17F, whereas brodalumab blocks IL-17 receptor A and therefore inhibits signaling mediated by several IL-17 family cytokines. These differences in target and mechanism may result in distinct efficacy and safety profiles (82). During treatment, patients should be monitored for mucocutaneous candidiasis and other class-related adverse effects of IL-17 inhibition, as well as paradoxical inflammation or shifts in disease phenotype (83, 84). In SAPHO syndrome, high-quality prospective controlled studies are needed before these agents can be reliably compared or positioned within a standardized treatment algorithm (15).

### 5.4 Targeting JAK/TYK2 and TNF signaling

JAK inhibitors can simultaneously interfere with multiple cytokine signaling pathways and have shown potential in the treatment of pustular psoriasis, nail psoriasis, associated joint disease, and osteitis-related pain in SAPHO syndrome (85–87). Their broad pathway coverage may be useful in patients with multisystem involvement, but it also makes it difficult to attribute clinical benefit to a specific signaling node (59, 71). Treatment requires individualized assessment and long-term monitoring for infection, hematological abnormalities, dyslipidemia, cardiovascular events, and thromboembolic risk (71, 88, 89).

Selective TYK2 inhibitors modulate IL-23, IL-12, and type I interferon signaling and have emerged as an oral targeted treatment option for psoriasis (90, 91). Their potential role and appropriate use in SAPHO syndrome, however, remain uncertain (24, 92, 93).

TNF-α inhibitors can improve refractory osteoarticular inflammation in some patients with SAPHO syndrome (2, 94, 95), but may also induce or worsen psoriasiform eruptions and paradoxical PPP (15, 51, 96). This discordant response has practical implications: the same therapy may improve osteitis while aggravating pustular skin disease, supporting the view that cutaneous and skeletal lesions do not depend on fully overlapping immune pathways (59, 83, 97).

### 5.5 Interpreting treatment responses and defining outcomes

Treatment selection should take into account the patient’s predominant clinical concerns, previous treatment history, comorbidities, the organs involved, and the strength of the available evidence. For patients with acute GPP and systemic inflammation, IL-36-targeted therapy may be prioritized when an approved agent is available and the indication is appropriate.

In patients with chronic PPP accompanied by inflammatory arthritis or SAPHO-like osteitis, treatment should address pathways relevant to both cutaneous and musculoskeletal disease. At present, however, the choice among IL-17 inhibitors, IL-23 inhibitors, TNF-α inhibitors, JAK inhibitors, and other therapies cannot be reduced to a standardized biomarker-based algorithm (1).

Outcome assessment should extend beyond short-term pustule clearance or pain relief. Studies of GPP should include time to pustule resolution, control of systemic inflammation, organ damage or failure, recurrence, and hospitalization. PPP and SAPHO syndrome require longer follow-up, with outcomes that capture palmoplantar pain, joint function, work capacity, quality of life, chest and back pain, MRI-defined bone marrow edema, and CT-defined structural progression. Composite outcome measures may be useful, but they have not yet been routinely adopted in clinical studies.

Treatment responses may also generate mechanistic hypotheses. Concurrent improvement in cutaneous and osteoarticular manifestations after IL-23/Th17 blockade would support the presence of shared downstream inflammatory pathways. A predominantly cutaneous response to IL-36 inhibition would suggest tissue-specific pathway dependence, whereas the development of new-onset PPP during TNF-α inhibition despite improvement in osteitis would point to divergent pathway effects across tissues (98).

No single treatment-response pattern is sufficient to establish taxonomic equivalence between diseases. **Table 2** summarizes the organ-specific effects of the major therapeutic pathways and the main limitations of the available evidence.

**Table 2:** Organ-specific treatment responses and strength of evidence across SAPHO syndrome and pustular skin diseases.

Therapy/pathway	Representative agents or interventions	Cutaneous response	Osteoarticular response	Strength of evidence and major limitations
NSAIDs and bisphosphonates	NSAIDs; bisphosphonates	Insufficient for PPP, GPP, or ACH.	May reduce bone pain or osteitis activity in SAPHO.	Mainly case series and observational studies; improvement does not establish a shared skin–bone origin or halted structural progression.
Conventional systemic therapies	Antibiotics; glucocorticoids; methotrexate; cyclosporine; acitretin	Variable improvement in pustular lesions; relapse is common.	Inconsistent effects on osteitis and structural progression; skin and bone responses may diverge.	Heterogeneous evidence with frequent organ-specific responses.
IL-36 receptor inhibition	Spesolimab; imsidolimab	Rapid improvement in acute GPP supported by clinical trials.	Insufficient evidence of efficacy in SAPHO.	Strongest evidence in GPP; use in SAPHO–pustular overlap remains exploratory.
IL-23/Th17 axis inhibition	IL-12/23 p40, IL-23 p19, IL-17A, IL-17A/F, and IL-17RA inhibitors	May improve psoriatic or pustular lesions, including PPP.	Reported improvement in some SAPHO-PPP or pustulotic arthro-osteitis cases.	Mainly case reports and observational studies in SAPHO; supports downstream convergence, not identical initiation. Monitor candidiasis and paradoxical or shifted phenotypes.
TNF-α inhibition	Adalimumab; infliximab; etanercept	Unpredictable; may induce or worsen psoriasiform eruptions or paradoxical PPP.	May improve refractory osteoarticular inflammation or bone pain in SAPHO.	Mainly observational; skin and bone responses may diverge.
JAK inhibition	Upadacitinib; abrocitinib	Preliminary benefit in pustular psoriasis, nail psoriasis, and related lesions.	Possible benefit reported in SAPHO case reports.	Mechanistic attribution is limited; assess infection, hematological, lipid, cardiovascular, and thromboembolic risks.
Selective TYK2 inhibition	Deucravacitinib and other selective TYK2 inhibitors	Promising oral strategy; evidence mainly from psoriasis.	Unestablished for SAPHO osteitis or hyperostosis.	No validated SAPHO treatment sequence; coordinated skin–bone response data are required.

NSAIDs, non-steroidal anti-inflammatory drugs; PPP, palmoplantar pustulosis; GPP, generalized pustular psoriasis; ACH, acrodermatitis continua of Hallopeau; SAPHO, synovitis, acne, pustulosis, hyperostosis, and osteitis.

## 6 Disease classification and a multidimensional research framework

### 6.1 Competing classification models

Classification of these conditions has traditionally followed the predominant organ involved. Pustular skin diseases are defined mainly by their cutaneous phenotype, whereas SAPHO syndrome is recognized through its characteristic osteoarticular manifestations, with or without accompanying skin disease (19). The identification of shared inflammatory pathways has challenged this organ-based separation, but pathway overlap alone is insufficient to justify a unified disease classification.

Three models can be considered from presented evidence: 1) the independent disease model, which emphasizes the distinctive skeletal phenotype of SAPHO syndrome, including chronic aseptic osteitis, hyperostosis, anterior chest wall involvement, and pathological bone remodeling (14, 20, 76); 2) the PPP-centered bridging model, which is based on the reproducible association between PPP and osteoarticular inflammation, including the established clinical concept of pustulotic arthro-osteitis (28); and 3) the broader spectrum model, which places SAPHO syndrome within a group of neutrophil-driven inflammatory disorders that converge on IL-36- and IL-23/Th17-related pathways (34).

Of these interpretations, the PPP-centered bridging model is the most consistent with the available evidence. It recognizes a clinically meaningful SAPHO–PPP overlap without assuming that SAPHO syndrome, GPP, ACH, and other pustular diseases share the same origin. The model also preserves the characteristic skeletal identity of SAPHO syndrome while allowing shared downstream inflammatory mechanisms to be investigated across diagnostic boundaries.

### 6.2 A multidimensional research framework

For research purposes, multidimensional phenotyping may be more informative than the use of a single diagnostic label. Relevant dimensions include the cutaneous phenotype, the anatomical distribution of musculoskeletal disease, imaging measures of inflammatory activity and structural damage, genetic and immunological features, and organ-specific treatment responses. Cutaneous variables should distinguish PPP, GPP-like systemic flares, acne, ACH, nail involvement, and treatment-induced pustular eruptions. Musculoskeletal variables should separately record anterior chest wall osteitis, axial involvement, peripheral arthritis, enthesitis, and structural bone remodeling.

Combining these dimensions may help identify research subgroups characterized by predominant pustular inflammation, PPP with osteoarticular involvement, axial osteitis, or treatment-associated phenotype switching. Such subgroups could be used to compare tissue signatures and organ-specific therapeutic responses across conventional diagnostic categories. They are not currently validated for diagnosis or treatment selection and should remain confined to hypothesis-driven research until supported by prospective studies.

## 7 Discussion

### 7.1 Main synthesis

The relationship between SAPHO syndrome and pustular skin diseases is better explained by partial convergence than by complete taxonomic equivalence. Clinical co-occurrence is the most evident for SAPHO syndrome and PPP, whereas evidence connecting SAPHO syndrome directly with GPP or ACH is much less developed. At the mechanistic level, neutrophil recruitment and IL-23/Th17- and IL-36-related signaling provide plausible points of convergence, but the strength and tissue specificity of the supporting evidence differ considerably among diseases.

The framework proposed here separates three questions that are often conflated: whether two phenotypes coexist in the same patients, whether they share downstream inflammatory pathways, and whether they arise from the same initiating disease process. Current evidence supports the first two propositions most clearly at the SAPHO–PPP interface, but remains insufficient for the third. **Figures 1** and **2** should be interpreted as evidence-organizing and hypothesis-generating models rather than representations of an established common pathogenesis.

### 7.2 Strength and hierarchy of evidence

The evidence base in this field is markedly uneven. GPP is supported by disease-specific genetic studies and clinical trials of targeted therapies, with particularly strong evidence for the IL-36 pathway. Epidemiological and genetic data for PPP are increasing, although its nosological position remains debated. More recently, integrated single-cell RNA sequencing and spatial profiling of lesional and non-lesional PPP skin identified IL-36G-expressing inflammatory keratinocytes and a regulatory Th17-like population coexpressing FOXP3, IL-17F, and IL-26. Spatial analysis further demonstrated preferential proximity between these lymphocytes and IL-36G-positive keratinocytes, providing direct tissue-level evidence for an IL-17/IL-36-associated inflammatory network in PPP (99). Comparable published single-cell or spatial data from SAPHO osteitis lesions remain extremely limited. By contrast, because SAPHO syndrome is rare and clinically heterogeneous, most available evidence comes from retrospective cohorts, imaging studies, small case series, and case reports. Direct comparisons between SAPHO bone lesions and pustular skin lesions from patients with PPP or GPP remain scarce.

Accordingly, the most reliable conclusions are phenotypic rather than causal. SAPHO syndrome and PPP may coexist in the same patient, cutaneous and skeletal inflammatory activity may follow different time courses, and the structural bone changes of SAPHO syndrome differ from those usually associated with pustular psoriasis. Evidence remains insufficient to support a single IL-36-centered mechanism, biomarker-defined SAPHO treatment subgroups, or a unified pathway-based therapeutic algorithm. Findings from periodontitis, hidradenitis suppurativa, spondyloarthritis, osteoarthritis, animal models, and *in vitro* systems may inform hypothesis generation, but they should not be treated as disease-specific evidence for SAPHO syndrome or pustular psoriasis.

From an evidence-hierarchy perspective, the SAPHO–PPP clinical overlap and the asynchronous behavior of skin and bone lesions are relatively consistent observations. IL-23/Th17, TNF-α, and neutrophil-related pathways support downstream mechanistic convergence, but not a shared initiating cause. By contrast, direct IL-36-mediated induction of SAPHO osteitis, molecularly defined SAPHO treatment subtypes, and a unified targeted treatment pathway remain hypotheses requiring prospective and tissue-resolved validation.

### 7.3 Alternative explanations

Several observations permit alternative interpretations outside a unified disease-spectrum model. First, although PPP is the most frequently reported cutaneous phenotype in SAPHO cohorts, it is not present in all patients, and cutaneous and skeletal disease activity often follow asynchronous courses (3, 14, 19, 66). PPP may therefore represent a closely associated phenotype in some patients but a comorbidity in others. Second, smoking is strongly associated with PPP (38). *C. acnes* has been proposed as a possible microbial contributor or disease-modifying factor in a subset of patients with SAPHO syndrome, although its causal role remains uncertain (43, 44, 58). Treatment-related phenotype shifts, including paradoxical pustular skin reactions during TNF-α inhibition, have also been reported (98). These factors may promote inflammation through different mechanisms and therefore do not necessarily indicate a single initiating cause. Third, much of the available evidence originates from single-center or observational cohorts, in which patients with complex multisystem manifestations may be overrepresented (65, 66). Referral and selection bias therefore cannot be excluded when estimating the frequency of SAPHO–PPP overlap. Finally, responses to broad immunomodulatory therapies may reflect inhibition of shared downstream pathways rather than correction of a common initiating defect (51, 59, 71). Discordant or paradoxical skin and osteoarticular responses during targeted treatment support this possibility (51, 83, 98).

These alternative explanations do not invalidate the overlap model, but they raise the evidentiary threshold required to support it. A convincing disease-spectrum model should predict clinically meaningful patient subgroups, reproducible molecular features, and differential treatment responses more accurately than conventional diagnostic classifications.

### 7.4 Limitations of this review and the available evidence

The principal limitation of this review is the heterogeneity and indirectness of the available evidence. Several mechanistic propositions discussed in this review are extrapolated from related inflammatory diseases, animal models, or *in vitro* experimental systems rather than from direct analyses of SAPHO osteitis lesions or paired skin and bone tissues from the same patients. We have therefore specified the experimental origin of such evidence where relevant and interpreted it as supporting biological plausibility rather than establishing disease-specific causality.

In addition, this narrative review does not provide quantitative estimates of disease prevalence, strength of association, or treatment effectiveness. The included studies were heterogeneous in diagnostic criteria, disease definitions, imaging methods, outcome measures, and study populations. Treatment evidence in SAPHO syndrome is particularly vulnerable to publication bias because most reports consist of retrospective cohorts, small case series, or successful individual cases, whereas treatment failures and discordant organ responses may be underreported.

The classification of PPP also varies across regions and nosological systems, complicating comparisons among PPP, GPP, and plaque psoriasis. In addition, neither cytokine profiles nor genetic variants have been validated as broadly applicable biomarkers for treatment selection in SAPHO syndrome or SAPHO–pustular overlap phenotypes.

Treatment responses should likewise be interpreted cautiously. Broad-acting therapies affect multiple signaling pathways, and apparent clinical improvement may be influenced by concomitant treatment, spontaneous fluctuation in disease activity, or differences in the timing of outcome assessment. Cutaneous lesions, pain, inflammatory activity, and structural bone changes may improve at different rates. Treatment-response observations should therefore be interpreted as hypothesis-generating evidence, particularly when cutaneous, inflammatory, and structural skeletal outcomes have not been assessed separately.

### 7.5 Testable hypotheses and future research

Future studies should focus on questions that can distinguish among the competing classification models. Prospective multicenter registries should apply standardized definitions of SAPHO syndrome, PPP, GPP, ACH, psoriatic arthritis, and pustulotic arthro-osteitis, while systematically recording the temporal relationship between cutaneous and skeletal manifestations. Standardized imaging protocols are also needed to distinguish inflammatory activity from structural damage. Cutaneous disease activity, pain, physical function, MRI-defined inflammation, and CT-defined structural progression should be assessed as separate outcomes.

Paired tissue studies will be particularly important. Single-cell RNA sequencing, spatial transcriptomics, proteomics, and immune receptor repertoire profiling could be applied to pustular skin lesions, entheses, synovium, and osteitis lesions to determine whether similar myeloid, neutrophil, and lymphocyte populations recur across organs. Comparative cohorts should include patients with SAPHO syndrome with and without PPP, isolated PPP, GPP, psoriatic arthritis, and non-inflammatory controls. The identification of shared immune-cell states together with bone-specific stromal programs in the SAPHO–PPP overlap subgroup would provide direct support for the overlapping-network model.

Mechanism-driven clinical trials should prespecify organ-specific hypotheses. For example, IL-36 inhibition could be evaluated for rapid control of pustular disease while osteitis is assessed independently by MRI. Studies of IL-17 or IL-23 blockade should examine whether cutaneous and skeletal responses are concordant or dissociated. JAK or TYK2 inhibitors could also be investigated alongside biomarker panels designed to identify treatment-responsive inflammatory states. Negative and discordant results should be reported in full because they may be as informative for disease classification as positive responses.

Digital imaging and machine-learning approaches may assist lesion detection and longitudinal phenotyping, but their use should be grounded in standardized clinical definitions and validated outcome measures. The immediate priority is not greater technical complexity, but the generation of reproducible, directly comparable evidence with high tissue-level resolution.

## 8 Conclusion

SAPHO syndrome and pustular skin diseases share certain clinical and immunological features, but this overlap is confined to specific phenotypic subgroups and does not establish that they represent a single disease entity. The association between SAPHO syndrome and PPP is supported more strongly than any direct relationship between SAPHO syndrome and GPP. IL-36 signaling, the IL-23/Th17 axis, neutrophil activation, keratinocyte dysfunction, and myeloid cell responses may represent shared inflammatory nodes. However, aseptic osteitis, hyperostosis, preferential anterior chest wall involvement, and chronic pathological bone remodeling remain characteristic features of SAPHO syndrome.

Taken together, current evidence is best explained by a model of overlapping inflammatory networks with tissue-specific outcomes and substantial etiological heterogeneity. This framework supports interdisciplinary disease recognition and mechanism-oriented research, while also underscoring that proposed molecular subgroups and targeted treatment strategies require validation in high-quality prospective studies before they can guide routine clinical practice.
